# Comparison of vaginal and abdominal hysterectomy:A prospective non-randomized trial

**DOI:** 10.12669/pjms.304.4436

**Published:** 2014

**Authors:** Bing Chen, Dong-Ping Ren, Jing-Xuan Li, Chun-Dong Li

**Affiliations:** 1 Bing Chen, Department of Obstetrics and Gynaecology, Air Force General Hospital, PLA, Beijing 100142, China.; 2 Dong-Ping Ren, Department of Obstetrics and Gynaecology, Air Force General Hospital, PLA, Beijing 100142, China.; 3 Jing-Xuan Li, Department of Obstetrics and Gynaecology, Air Force General Hospital, PLA, Beijing 100142, China.; 4 Chun-DongLi, Department of Obstetrics and Gynaecology, Air Force General Hospital, PLA, Beijing 100142, China.

**Keywords:** Abdominal hysterectomy, Gynaecological benign diseases, Laparotomy, Vaginal hysterectomy

## Abstract

***Objective: ***To compare outcomes of vaginal and abdominal hysterectomy procedures in women with benign gynaecological diseases.

***Methods: ***This was a prospective study of outcomes of consecutive patients who underwent total vaginal hysterectomy (VH) or abdominal hysterectomy (AH) for benign gynaecological diseases. Patient characteristics before, during, and after the operations were reviewed. Patients were followed up for three months to evaluate postoperative complications.

***Results:*** This study included a total of 313 patients. 143 patients underwent AH and 170 patients underwent VH. Baseline characteristics were similar between the two groups. There were no intraoperative complications in either group. Operation time, intraoperative blood loss, first postoperative flatus time, time to out-of-bed activity, mean maximum postoperative body temperature, and duration of fever were all significantly shorter and less severe in the VH group compared with the AH group. In addition, vaginal length in the VH group was significantly shorter than in the AH group.

***Conclusions: ***Vaginal hysterectomy has advantages over AH in the treatment of benign gynaecological diseases, providing greater efficacy and safety with minimal invasiveness.

## INTRODUCTION

Hysterectomy is currently one of the most common gynaecological surgical procedures.^1^ In the United States, hysterectomy is second to Caesarean delivery as the most frequently performed major surgical procedure for women of the reproductive age. Approximately one in three women has undergone a hysterectomy by age 60, with approximately 600 000 hysterectomies performed annually in the United States.^2^

Routes for hysterectomy include abdominal, vaginal, laparoscopic, or combined approaches. Traditional abdominal hysterectomy (AH) is one of the most common gynaecological surgical procedures in the treatment of benign gynaecological diseases. However, AH as the most invasive procedure, is associated with some limitations such as abdominal trauma, intraoperative and postoperative complications, and slow postoperative recovery.^3^ Compared with traditional open gynaecological surgeries, minimally invasive gynaecological surgery provides less postoperative pain, more rapid recovery, and shorter hospital stay.^4^ Vaginal hysterectomy (VH) is the method of choice for removal of the uterus in patients with benign gynaecological diseases.^5^ For patients of advanced age and small uterus size, the VH procedure has some advantages over AH procedure, including less complications, shorter hospital stay, and faster recovery.^6^ According to the surveillance data from 1995-1996 in the UK, most hysterectomies in the UK are abdominal (70–90%) with only 10–30% performed vaginally and less than 5% laparoscopically.^7^A recent report in Denmark shows that the use of VHs increases from 12 to 34%, accompanied with a decrease in the use of AH.^8^ However, VH is contraindicated in patients with large uterine size^9^, because the vaginal route offers relatively limited space for surgical procedure. Therefore, surgeons have greater confidence in operating through the abdominal route if adequate surgical haemostasis is maintained.

Surgical haemostasis can be secured by a variety of methods, including mechanical (sutures) or vessel coagulation (diathermy), although electrocoagulation diathermy has been shown to be unreliable for vessels larger than 2 mm in diameter.^10^ Electrosurgical vessel sealing is a new haemostatic system based on the combination of pressure and bipolar electrical energy, and is able to seal vessels up to 7 mm in diameter. This sealing system has been used in VH with encouraging results.^11^^,^^12^ The LigaSure™ system is a common electrosurgical vessel sealing system used in VH, and has been shown to reduce complications in comparison to AH.^13^

The present study examined outcomes of patients who underwent VH or AH at the Department of Obstetrics and Gynaecology, Air Force General Hospital, PLA, Beijing, China. The purpose of this study was tocompare the feasibility and safety of VH and AH procedures in the treatment of benign uterine diseases, and to determine the outcomes of both procedures.

## METHODS

***Patients*** ***: ***This prospective study included 313 female patients who underwent VH or AH at the Department of Obstetrics and Gynaecology, Air Force General Hospital, PLA, Beijing, China, from January 2005 to December2008. All patients underwent physical, ultrasound, and biopsy examinations. All cases were diagnosed with uterine benign diseases, including uterine fibroids, adenomyosis, cervical intraepithelial neoplasia (CIN) III, and endometrial atypical hyperplasia. Patients were diagnosed based on clinical symptoms and signs and ultrasound examinations, and confirmed by biopsy examinations. All patients were followed up for at least 3 months. Inclusion criteria were: 1) uterine benign diseases such as uterine fibroids, adenomyosis, and CIN; 2) gynecological symptoms that justified total hysterectomy; 3) indicated for either AH or VH; 4) patients without fertility requirement; 5) patients with the follow-up period of more than 3 months; and 6) patients who gave their informed consent to participate. Exclusion criteria were: 1) malignant cancers; 2) uterine size equivalent to > 12 weeks gestation; 3) nulliparity or no prior vaginal delivery; 4) previous Caesarean delivery; 5) desire to perform salpingo-oophorectomy; 6) patients with the follow-up period of less than 3 months; and 7) patients with fertility requirement.

The gynecologist allocated the patient to either AH or VH according to preferred clinical grounds. Patients were selected for AH with the following clinical criteria: 1) fixed uterus or no uterine descent; 2) unmarried women; and 3) vaginal stenosis. Patients were selected for VH with the following clinical criteria: 1) freely mobile uterus; and 2) more than one vaginal delivery.

Routine systemic, gynaecological, and cervical cytological examinations were performed for all patients who underwent total hysterectomies. Fractional curettage was performed to exclude gynaecological malignancies. Patient characteristics (e.g., age, weight, body mass index [BMI]), were recorded. The Ethical Research Committee of The Air Force General Hospital, PLA, Beijing, China, approved this study, all subjects gave their informed consent. All procedures were performed by the same surgeon (CL).

All patients underwent combined spinal-epidural anaesthesia (CSEA). Under sterile conditions, the epidural space at the L2-L3 level was entered using a loss-of-resistance technique, and a Tuohy needle. A 25G spinal needle was introduced via the Tuohy needle into the subarachnoid space. After the cerebrospinal fluid was seen dripping from the spinal needle, 1-2 ml of bupivacaine (0.75%) was injected. The spinal needle was then withdrawn, and an epidural catheter was threaded through the Tuohy needle into the epidural space. If the level of anaesthesia from the spinal anaesthetic was judged to be inadequate for the operation, lidocaine (2%) was injected via the epidural catheter.

***Surgical procedures***

***Vaginal hysterectomy: ***Before VH, a dilute solution (1:10,000) of adrenaline was injected into the submucosal tissues at the junction of the cervix and the vagina to circumcise mucous membranes, and was also injected into tissues at the junction of the cervix and the bladder to reduce intraoperative blood loss and clarified the boundaries of the cervix and surrounding tissue. The mucous membranes were then circumcised to the cervical fascia at the junction of the cervix and vagina. The edge of vaginal mucosa was lifted by tissue forceps. The bladder was then gently dissected from the vagina anteriorly, and the pouch of Douglas was opened posteriorly close to the cervical fascia. The uterus, bilateral uterosacral, cardinal ligaments, and uterine vessels were sealed using the LigaSure™ system (Covidien, Boulder, CO, USA) according to the manufacturer’s instruction. At the bend of peritoneum, the uterine anterior and posterior leaves of the visceral peritoneum were opened to reveal the uterine anterior and posterior leaves of the visceral peritoneum. The inherent ovarian ligament, round ligament and fallopian tube were then hooked and pulled down, followed by dissecting these ligaments and the fallopian tube using LigaSure™ system. For patients with a larger uterus, if it was difficult to remove,it was dissected into small pieces and removed. For patients with a small uterus, it was removed entirely, without dissection. If the uterus featured uterine fibroids, myomectomy would be considered first to reduce the volume of the uterus before the hysterectomy. To suture the basin peritoneum and vaginal stump, the pelvic peritoneum and the vaginal anterior and posterior walls of stump were sutured with absorbable sutures (Johnson & Johnson, Piscataway, NJ, USA). This process helped to reduce dead space and blood loss.

***Abdominal hysterectomy*** ***: ***A transverse incision was made across the lower abdomen. The uterus was pulled to expose the round ligament and adnexa. The ford of the uterine visceral peritoneum was cut, and the bladder was gently moved to the level of cervical external aperture. The uterine blood vessels were then clamped and ligated at the cervical internal aperture. The ends of the vessels were doubly ligated. At the level of cervical internal aperture, the cervix was circularly cut 3-4 mm under the cervical facia, and the uterus was removed, after the cervical fascia and its attached bladder were stripped off. A 1/0 micro-bridge suture line was used to close the ends of vagina. The peritoneum on the bilateral round ligament and the adnexal ends were sutured. The round ligament and the adnexal ends were embedded in the peritoneum followed by suturing of the anterior and posterior pelvic peritoneum. The abdominal cavity was then closed, and the skin and subcutaneous fat was sutured using a 4-0 mersilk line.

***Postoperative care*** ***: ***All patients were treated orally with cefuroxime (2.5 g twice a day) and ormidazole (1 g daily) 1 hour before and 1 day after the operation to prevent infection as postoperative routine measures. 20 ml of 0.75% bupivacaine, 100 µg sufentanil, and 3 mg granisetron in saline was infused via an analgesia pump to control postoperative pain in all patients.

***Outcomes*** ***: ***The following parameters were recorded: patient’s general information such as age and body weight, uterine size, operation time, intraoperative and postoperative complications, postoperative body temperature, postoperative vagina length, time to first postoperative flatus postoperation, postoperative pain, the number of days before out-of-bed activity, and duration of hospital stay. A numeric pain rating scale was used, in which patients rated their own pain using a scale of 0-10. It was measured 2, 12, and 24 hr after surgery. Postoperative vagina length was measured at the follow-up period of three months.

***Statistical analyses*** ***: ***All data were presented as the mean ± SE. The difference in means between groups was tested using an independent Student’s *t*-test and Wilcoxon’s rank–sum test. All statistical analyses were performed using SPSS^®^ version 10.0 (SPSS Inc., Chicago, IL, USA) and the SigmaStat software package (version 3.5, SPSS Inc., Chicago, IL, USA). A *P*-value < 0.05 was considered statistically significant.

## RESULTS

A total of 313 patients were included in the study. The mean patient age was 47.3 ± 6 years (range, 35-68 years). 170 patients underwent VH and 143 patients underwent AH. Patient characteristics are shown in Table-I. Patient age and body weight did not differ significantly between the VH and AH groups. Prior to surgery, the uterine sizes were equivalent to 8 – 16 weeks of gestation. The diseases were diagnosed by pathological examination, and the results are also shown in Table-I**.** The diseases in each group were comparable.

No case was converted to open surgery. There were no intraoperative complications such as bladder, rectum or urethra injuries in any groups. Compared with AH, VH was associated with a shorter mean operation time (VH: 65.2 ± 10.6 min, AH: 95.6 ± 15.9 min; P<0.05) and less mean intraoperative blood loss (VH: 30.4± 10.5 ml, AH: 70.3 ± 18.6 ml; *P *< 0.05) (Table-II).

Postoperative pain was not obvious in patients in each group due to the administration of analgesics. Mean time to first postoperative flatus, mean number of days before out-of-bed activity, and mean maximum postoperative body temperature in the VH group were significantly shorter and less severe than those in the AH group (*P*< 0.05). At the follow-up period of three months, the vaginal length in the VH group was significantly shorter compared with the AH group (*P *< 0.05, Table-II).

## DISCUSSION

Hysterectomy, the most common major surgical procedure for gynaecological conditions, is used for both malignant diseases and benign conditions such as fibroids, endometrial hyperplasia, adenomyosis, endometriosis, uterine prolapse, dysfunctional uterine bleeding, and cervical intraepithelial neoplasia.^14^ There are many approaches to hysterectomy for benign diseases, including AH, VH, laparoscopic assisted vaginal hysterectomy (LAVH), total laparoscopic hysterectomy (TLH), and subtotal laparoscopic hysterectomy. With the constant modernization of minimally invasive concepts in obstetrics and gynaecology, doctors choose surgical routes by considering not only the patient's health status, but also the psychological needs of patient and the patient quality of life after surgery. The choice between vaginal, laparoscopic or abdominal routes remains controversial.

Extensive studies have been performed to compare different hysterectomies. A comprehensive and systematic review compared AH and VH with laparoscopic hysterectomy, and assessed their potential beneficial and adverse effects in women with benign gynaecological conditions.^15^ Compared with AH, the beneficial effects of VH included shorter time to normal activities, fewer febrile episodes or unspecified infections, shorter duration of hospital stay, lower intraoperative blood loss, and fewer wound or abdominal wall infections.^15^ In addition, fewer febrile episodes or unspecified infection and shorter operation time were noticed in LAVH procedures compared with TLH procedures. LAVH is also preferred in patients with a mass in the lower segment or a relatively large uterus.^16^ Operation time and bleeding are increased in TLH as compared with LAVH.^17^ TLH is associated with greater safety, efficacy, and improvement in the patient quality of life compared to total AH in women with benign gynaecological diseases.^18^ TLH has been regard as a more cost-effective procedure, and has several advantages over total AH, such as smaller incision, less postoperative pain, shorter hospital stay, faster recovery time and less serious complications.^18^

**Table-I:** Demographics and clinical characteristics of patients who underwent vaginal hysterectomy (VH) or abdominal hysterectomy (AH).

	***VH *** ***(n = 170)***	***AH *** ***(n = 143)***
Age, years^a^	48 ± 6	46 ± 5
Weight, Kg^a^	57 ± 8	56 ± 9
Diagnosis, n		
Hysteromyoma	105 (61.8%)	95 (66.4%)
Adenomyosis	25 (24.7%)	20 (14.0%)
CIN	20 (11.8%)	15 (10.5%)
Atypical hyperplasia of endometrium	20 (11.8%)	13 (9.1%)

a Data presented as mean ± SE and *n* of patients. CIN, cervical intraepithelial neoplasia.

**Table-II:** Operative and postoperative outcomes in patients who underwent vaginal hysterectomy (VH) or abdominal hysterectomy (AH) for the treatment of benign gynaecological conditions

	***VH *** ***(n = 170)***	***AH *** ***(n = 143)***
Operating time (min)	65.2 ± 10.6*	95.6 ± 15.9
Blood loss (ml)	30.4 ± 10.5*	70.3 ± 18.6
Time to first postoperative flatus (h)	22.9 ± 4.0	35.5 ± 10.2
Time to out-of-bed activity (h)	23.1 ± 2.4	35.4 ± 4.3
Hospital stay (days)	4.5 ± 0.5	6.3 ± 1.5
Postoperative body temperature (°C)	37.4 ± 0.14*	38.3 ± 0.25
Postoperative vagina length (cm)	8.4 ± 0.9*	9.6 ± 0.8

a Data presented as mean ± SE.

* *P *< 0.05 compared with AH; Student’s *t*-test.

The route of hysterectomy is guided by the surgical indication for hysterectomy, patient anatomy, data that support the selected procedure, informed patient preference, and the surgeon’s expertise.^15^ The common indications for traditional VH include good uterine activity, volume of uterus equivalent to less than 12 weeks’ gestation, no history of pelvic surgery, normal adnexa, wide maternal pelvis, and no other anaesthetic or surgical contraindications. In this study, VH was performed in patients with uterine size equivalent to 8-16 weeks, and was associated with less operation time, less intraoperative blood loss and better postoperative outcomes compared with AH, suggesting that VH is an effective treatment for patients with benign gynaecological diseases. 

In addition, Mistrangelo *et al.* reported that VH was safe and effective in cases of greater uterine weight or volume.^19^ Guvenal *et al. *found that VH could be performed with less morbidity, even in patients with a large, immobile uterus and previous pelvic surgery.^20^ Falcone et al have confirmed the success of the vaginal approach in patients with these characteristics.^21^ Rates of urethral and bladder injuries at the time of VH were 0.88% and 1.76%, respectively.^22^ Consistent with this, in a recent large case series, the incidence of bowel injury was low in VH patients.^22^ Furthermore, conversion rates from the vaginal to abdominal approach have been reported to be of 0.4% in a retrospective review of 220 patients.^23^^-^^25^ In this study, no intraoperative complications occurred in patients of the VH group, and no vaginal approach was converted to an abdominal approach. Taken together, all these studies indicate that VH is a safe and effective surgical treatment for benign gynaecological diseases.

In summary, this study shows that the choice of hysterectomy procedure should be considered according to the patient’s disease, and the use of the most appropriate surgical equipment/devices. In general, to ensure the efficacy and safety of the operation, the most minimally invasive surgery should be chosen and VH appears to have advantages over AH in the treatment of benign gynaecological diseases.

## Author Contribution:

**BC:** Carried out the management of all patients, participated in all operations.

**DPR:** Participated in the design of the study and performed the statistical analysis.

**JXL: **Participated in some of the operations.

**CDL: **Conceived the study, participated in its design, carried out all the operations and coordination and helped to draft the manuscript. All authors have read and approved the final manuscript.

## Notes

***Conflict*** ***of interest: ***The authors declare that there is no conflict of interest.
